# Parkinson's disease and glucose metabolism impairment

**DOI:** 10.1186/s40035-025-00467-8

**Published:** 2025-02-17

**Authors:** Liangjing Chen, Chunyu Wang, Lixia Qin, Hainan Zhang

**Affiliations:** 1 Department of Neurology, Second Xiangya Hospital, Central South University, Changsha, 410011 China; 2 Clinical Medical Research Center for Stroke Prevention and Treatment of Hunan Province, Department of Neurology, Second Xiangya Hospital, Central South University, Changsha, 410011 China

**Keywords:** Parkinson's disease, Glucose metabolism, Mechanisms, Therapeutic drugs

## Abstract

Parkinson's disease (PD) is the second most common neurodegenerative disorder. PD patients exhibit varying degrees of abnormal glucose metabolism throughout disease stages. Abnormal glucose metabolism is closely linked to the PD pathogenesis and progression. Key glucose metabolism processes involved in PD include glucose transport, glycolysis, the tricarboxylic acid cycle, oxidative phosphorylation, the pentose phosphate pathway, and gluconeogenesis. Recent studies suggest that glucose metabolism is a potential therapeutic target for PD. In this review, we explore the connection between PD and abnormal glucose metabolism, focusing on the underlying pathophysiological mechanisms. We also summarize potential therapeutic drugs related to glucose metabolism based on results from current cellular and animal model studies.

## Introduction

Parkinson's disease (PD) is a common neurological disorder affecting middle-aged and elderly individuals. Clinically, it is characterized by resting tremor, bradykinesia, muscle rigidity, and postural instability [1]. Most PD cases are sporadic, and it is reported that 5% to 10% of PD may be caused by a single pathogenic mutation (single gene). Other causes of PD include a combination of complex genetic susceptibility and environmental factors [2]. The primary pathological changes in PD involve the degeneration and death of dopaminergic (DA) neurons in the substantia nigra (SN) pars compacta [3]. However, the exact cause of this neuronal degeneration remains unclear. It is currently believed that PD results from interactions of multiple factors rather than a single cause. Recognized mechanisms include oxidative stress, mitochondrial dysfunction, neuroinflammation, insulin resistance, and protein misfolding. Recent human studies have highlighted presence of bioenergetic maladaptation in brains in aging and neurodegenerative disorders, including PD [4, 5].

**Fig. 1:**
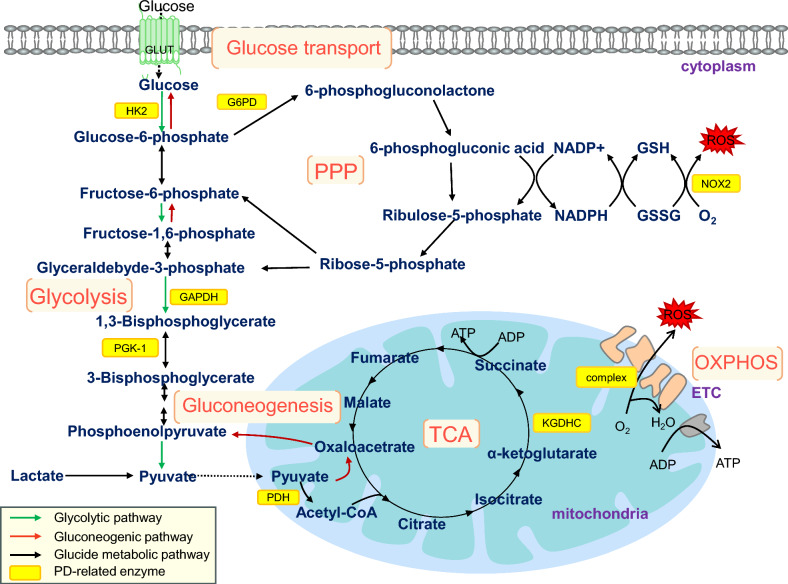
Mechanism of glucose metabolism and key enzymes related to PD. The process of glucose metabolism includes glucose transport, glycolysis, gluconeogenesis, TCA, oxidative phosphorylation and pentose phosphate pathway. Damage to key enzymes can lead to disorders of cell energy metabolism, resulting in the occurrence of PD. PPP, pentose phosphate pathway; OXPHOS, oxidative phosphorylation; TCA, tricarboxylic acid; GLUT, glucose transporter; HK2, hexokinase 2; PGK-1, phosphoglycerate kinase-1; PDH, pyruvate dehydrogenase; GAPDH, glyceraldehyde-3-phosphate dehydrogenase; G6PD, glucose-6-phosphate dehydrogenase; KGDHC, α-ketoglutarate dehydrogenase complex; ROS, reactive oxygen species; α-syn, α-synuclein; ETC, electron transport chain

Under normal physiological conditions, glucose is continuously supplied to the brain, providing nearly all the adenosine triphosphate (ATP) required by brain cells [6]. The physiological processes of glucose metabolism include glucose transport, glycolysis, tricarboxylic acid cycle (TCA), oxidative phosphorylation (OXPHOS), gluconeogenesis and pentose phosphate pathway (PPP) (Fig. 1). In addition, glucose enters the brain from the blood by crossing the blood–brain barrier through glucose transporter 1 (GLUT1). Glucose and other metabolites (e.g. lactate) are rapidly distributed through a highly coupled metabolic network of brain cells [7]. Glucose provides energy for neurotransmission [8], and several glucose-metabolizing enzymes control cell survival [9, 10]. Disturbed glucose metabolism on any of these levels can lead to development of a large variety of disorders in the brain.

It has been reported that PD and diabetes share a common pathway of disorders [11], and cohort studies have shown that diabetes is an independent risk factor for PD [12]. Therefore, glucose metabolism disorders in PD have been studied in more detail [13]. Abnormal glucose metabolites in cerebrospinal fluid (CSF) and functional imaging of cerebral glucose metabolism using fluorodeoxyglucose positron emission tomography (FDG-PET) hold promise for early and differential diagnosis of PD [14, 15]. In addition, antidiabetic drugs are gradually becoming a new treatment for PD [16]. In this review, we summarize the latest findings on glucose metabolism in PD and its clinical relevance, describe the underlying mechanisms by which abnormal glucose metabolism may contribute to PD, and outline potential therapeutic drugs that target glucose metabolism.

## The impact of glucose dysfunction on the risk and progression of PD

Glucose is the main circulating energy substrate for the adult brain. Glucose is actively oxidized to produce ATP to meet the high energy demand of nerve cells. Glucose has a synergistic effect with mitochondria in metabolic pathways. Dysfunction of glucose metabolism impairs the normal functioning of neurons, which is widely observed in neurodegenerative diseases [17].

### Glucose metabolism impairment and the risk of PD

Accumulating evidence from epidemiological studies suggests that hyperglycemia and diabetes may increase the risk of sporadic PD. An earlier meta-analysis of seven cohort studies (encompassing 1,761,632 patients) reported that diabetic patients have a 38% higher risk of developing PD compared to non-diabetic individuals, with the risk being 50% higher in women and 40% higher in men [18]. Beyond that, impaired glucose tolerance found in prediabetic patients, can also modify PD risk to some extent. For example, a retrospective cohort study of prediabetic patients (Hemoglobin A1c ≥ 5.7%–6.4%, no use of antidiabetic drugs, and no prior diagnosis of type 2 diabetes mellitus (T2DM)) indicated that prediabetes is associated with an increased risk of PD, with a hazard ratio of 1.07 (95% confidence interval: 1.00–1.14) [19]. Similarly, a recent meta-analysis of 15 cohort studies with over 86,000 PD cases and nearly 30 million subjects, found that prediabetic patients have a 4% increased risk of PD compared to individuals with normal blood glucose level [20]. More details of research are summarized in Table 1.

**Table 1:** Epidemiological studies on the associations between diabetes and PD

Year	Country	Study design	Sample size	Outcomes	Reference
2011	USA	Prospective	*n* = 288,662 Diabetes: 21,611 Non-diabetic: 267,051 PD: 1565	T2DM increases the risk of PD by 40%	[21]
2012	China	Retrospective	*n* = 1,075,604 T2DM: 603,416	T2DM is associated with an increased risk of PD	[22]
2012	Italy	Case–control	*n* = 961 PD: 783 T2DM: 89	T2DM onset before PD is associated with higher UPDRS motor scores and higher Hoehn and Yahr staging	[23]
2017	China (Taiwan region)	Case–control	*n* = 145,176 T2DM: 36,294	The incidence density rate of PD is 1.36-fold higher in T2DM than in non-diabetic controls	[24]
2017	Spain	Case–control	*n* = 4998 PD: 79	The prevalence of PD is positively correlated with long T2DM duration	[25]
2018	UK	Prospective	*n* = 6,173,208 T2DM: 2,017,115	T2DM increases the risk of PD. A greater increase is observed in those with complicated T2DM	[26]
2018	USA	Case–control	*n* = 53 PD-T2DM: 25 T2DM: 14 HC: 14	T2DM is associated with faster motor progression and cognitive decline	[27]
2020	Korea	Retrospective	*n* = 8,443,351 IFG: 2,110,252	IFG is associated with an increased risk of PD	[12]
2020	UK	Case–control	*n* = 501,780 PD: 1276	T2DM is associated with an increased risk of PD	[28]
2021	Catalonia, Spain	Retrospective	*n* = 2,556,928 T2DM: 281,153 Prediabetes: 266,379	T2DM and prediabetes are associated with an increased risk of PD	[19]
2021	Austria	Cross-sectional	*n* = 2,173,441 PD: 235,268	Men with T2DM have an increased risk of PD than non-diabetic men. Women with T2DM have a greater risk of PD than men with T2DM	[29]
2022	UK	Case–control	*n* = 1930 PD-T2DM: 167 PD: 1763	T2DM patients have more severe motor symptoms assessed by UPDRS-III and non-motor symptoms scales	[30]
2022	Italy	Retrospective and prospective	*n* = 9862 PD: 8380 PD-T2DM: 673	Occurrence of T2DM before PD onset is associated with a worse prognosis	[31]

IFG, impaired fasting glucose; HR, hazard ratio; OR, odds ratio; RR, risk ratio

As mentioned above, most studies support a close relationship between PD risk and hyperglycemia. Systemic glucose metabolism impairment may also increase the risk of PD.

### Impaired glucose metabolism and PD progression

The typical symptomatology of PD includes motor symptoms, such as tremor at rest, rigidity, and bradykinesia [1]. Some clinical studies indicate that impaired glucose metabolism can exacerbate the progression of sporadic PD. In Cox survival analyses of PD patients, diabetes is associated with faster disease progression (hazard ratio = 4.521, 95% confidence interval = 1.468–13.926; *P* < 0.01) [27]. Furthermore, PD patients with diabetes exhibited higher subscores for postural instability and gait difficulties after controlling for dopaminergic denervation in the SN and striatum (*t* = 3.81, *P* = 0.0005) [32]

Apart from motor symptoms, PD is characterized by a wide variety of non-motor features, including dementia, depression, sleep disturbance, orthostatic hypotension, and psychotic symptom [1]. Some studies have shown that diabetes is associated cognitive impairment in patients with PD [33]. For example, a study showed that compared to non-diabetic PD patients, PD patients with diabetes have significantly lower average overall cognitive Z scores (− 0.98 ± 1.01 vs − 0.36 ± 0.91). Importantly, diabetic and non-diabetic PD subjects show significant differences in SN and striatum denervation or cortical cholinergic denervation, as assessed by PET imaging. ANCOVA analysis demonstrated that diabetes is an independent factor that influences cognitive impairment in PD [34].

Consequently, PD patients with diabetes typically experience more severe motor symptoms and cognitive impairments. However, most of these studies employed small sample sizes. Larger-sample studies are needed to verify these findings.

### FDG-PET

FDG-PET is an imaging approach that reflects the molecular metabolism of glucose, using ^18^F as the marker nuclide. At the early stage of molecular-level change before the presence of morphological lesions, FDG-PET can detect molecular metabolism changes. A large number of studies have used FDG-PET to confirm the presence of abnormal glucose metabolism in brains of PD patients. In 2009, one study using FDG-PET found that PD patients had significantly higher normalized brain metabolic rates of glucose in the bilateral globus pallidus and SN, contralateral caudate nucleus, and ipsilateral putamen relative to the clinically most affected body side, compared to healthy subjects [35]. Likewise, a study utilizing FDG-PET imaging of brain glucose metabolism revealed that PD is characterized by hypermetabolism in the putamen, pallidum, thalamic sensorimotor cortex, pons, and cerebellum, along with relative hypometabolism in the posterior temporal-parietal lobe, occipital lobe, and occasionally the frontal lobe, particularly in PD patients with cognitive impairment [36].

To better explain the association between PD dysfunction and metabolic changes in brain regions, several PD-related patterns have been derived. The PD cognition-related pattern is represented by relative hypometabolism in the frontal and parietal association areas with concurrent relative hypermetabolism in the cerebellar vermis and dentate nuclei [37]. Similarly, the PD-specific pattern (PDSP) in an adeno-associated viral vector (AAV)-based α-synuclein (α-syn) rat model of PD indicated that except entorhinal, insular and ipsilateral somatosensory cortex, all cortical regions, as well as striatum, midbrain, pons and medulla oblongata, have metabolic hyperfunction, while thalamus, hippocampus and cerebellum have metabolic hypofunction. In addition, the longitudinal PDSP expression scores are significantly different between the PD group and the control group at time points consistent with the pathophysiology of the animal model (4, 6, and 9 weeks after AAV injection). These findings indicate that the metabolic changes reflected by PDSP reflect PD-related pathogenesis [38].

Impaired glucose metabolism may also reflect cognitive impairment in PD patients. FDG-PET imaging has revealed significant metabolic declines in the middle frontal lobe and lower parietal lobe of PD patients with mild cognitive impairment, compared to those without mild cognitive impairment [39]. Reduced performance in memory-based tasks is associated with decreased FDG metabolism in the posterior parietal and temporal lobe regions, while attention performance is associated with more frontal lobe deficits [40]. Furthermore, in advanced PD, significant decreases of glucose metabolism have been observed in the bilateral precuneus (Brodmann area 31), left middle temporal gyrus (Brodmann area 21), and left fusiform gyrus (Brodmann area 37), which may be associated with notable cognitive decline [41]. Interestingly, the transition from cognitively normal PD, through mild cognitive impairment, to PD dementia (PDD) is marked by a decrease in parietal-occipital metabolism, which may be an early predictor of dementia in PD [42]. Besides, visual hallucination (VH) is a common complication and a risk factor for PDD. Compared to VH-negative patients, PD patients with VH also exhibit lower bilateral FDG uptake in the occipital lobe, parietal cortex, right temporal lobe, and left cingulate, indicating a more severe brain metabolic decline and a higher risk of progression to dementia [43].

Idiopathic rapid eye movement sleep behavior disorder (iRBD) represents a well-known precursor phase of PD, in which pathology is thought to have reached the lower brainstem [44]. The majority of iRBD patients will develop into apparent PD within a few years to decades. In a longitudinal study, regional FDG-PET brain changes were analyzed in 25 iRBD patients at baseline, and after 2 and 4 years. The PD converters had greater metabolic increases in the bilateral putamen and metabolic decreases in the premotor cortex, superior frontal gyrus and supplementary motor area over time [45]. iRBD patients have shown significant changes over time at both individual and population levels, which may represent a functional compensation for ongoing neurodegeneration.

## Glucose metabolism impairment in PD

### PD leads to glucose metabolism impairment

PD may lead to glucose metabolism disorders through mechanisms such as intestinal microbial disorders, autonomic nerve dysfunction, abnormal insulin signal transduction and decreases of dopamine receptors. A previous study has found significantly increased *pseudoflavonifractor* in the gut of PD patients compared to the healthy control group [46]. *Pseudoflavonifractor* is an intestinal microorganism associated with energy metabolism and insulin sensitivity [47], and its high abundance is closely related to metabolic disorders [48].

In another study, non-diabetic PD patients showed higher blood glucose levels after glucose tolerant test compared to healthy matched controls, but did not show the expected increase in plasma insulin at the same time. Simultaneously, the higher blood glucose levels were significantly associated with higher score of dysautonomia [49]. The results suggest impaired insulin response to high glucose levels in PD, which may be due to the failure of β cells to enhance insulin secretion to cope with elevated blood glucose. Interestingly, the proliferation and function of β cells are regulated by the autonomic nervous system [50], and autonomic dysfunction is a well-known feature of PD [51]. Thus, autonomic dysfunction in PD may lead to β cell dysfunction and insufficient insulin levels to cope with high glucose levels.

Additionally, brain glucose metabolism may be controlled by neuronal insulin and its receptor signaling pathways [52]. A study in 1996 analyzed the mRNA levels of insulin receptors in the SN of postmortem PD human brains and detected reduced insulin receptor mRNA compared to the control brains [53]. Therefore, abnormal insulin signal transduction in PD patients can also lead to cerebral glucose metabolism disorders.

**Fig. 2:**
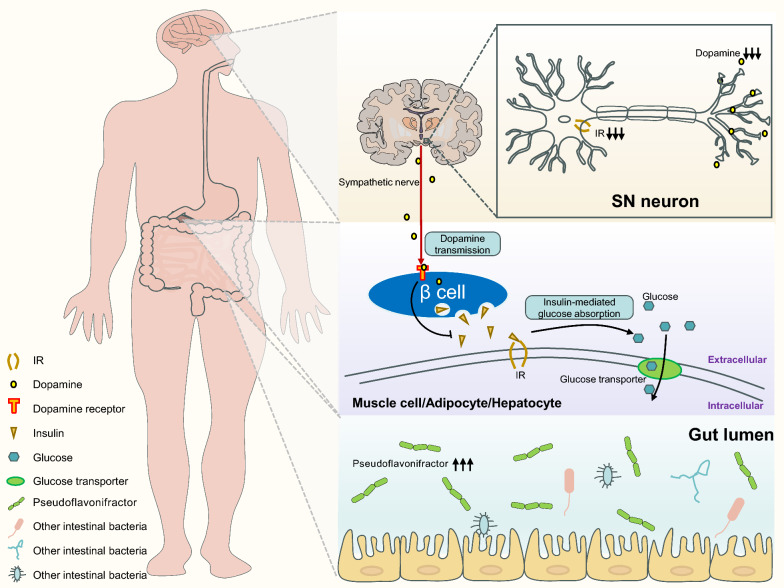
Possible mechanisms of glucose metabolism impairment caused by PD. Firstly, the degeneration and loss of DA neurons in the SN of PD patients results in decreased dopamine secretion. Additionally, the reduction of insulin receptor expression in the SN and abnormalities in insulin signaling pathways contribute to disrupted glucose metabolism in the brain. Secondly, reduced dopamine release from sympathetic nerve endings affects dopamine receptors on pancreatic β cells, inhibiting insulin secretion. This, in turn, impairs glucose utilization in muscle, adipose tissue, and hepatocytes, leading to systemic glucose metabolism disorders. Thirdly, *Pseudoflavonifractor*, a gut microorganism associated with energy metabolism and insulin sensitivity, shows an abnormal increase in PD patients, potentially exacerbating glucose metabolism impairment. DA, dopaminergic; SN, substantia nigra; IR, insulin receptor

A functional brain imaging study of 63 elderly subjects found increased insulin resistance in the brains of PD patients [54]. Studies in rodent models have shown that insulin resistance may lead to decreased expression of surface dopamine transporters in the striatum [55], decreased dopamine turnover [56], and decreased insulin-dependent dopamine release in the striatum [57]. In recent years, accumulating evidence shows that dopamine is key to glucose homeostasis. In PD, the degeneration and loss of DA neurons in the SN lead to reduced dopamine secretion in the brain. Dopamine affecting pancreatic β cells is derived either from neurons innervating the pancreatic islets or from the pancreas itself. Sympathetic nerve terminals release dopamine, which binds to dopamine receptors on β cells, thereby inhibiting insulin secretion [58]. Therefore, decreases of dopamine in PD patients may lead to glucose metabolism disorders (Fig. 2).

### Glucose metabolism impairment is involved in the pathogenesis of PD

**Fig. 3:**
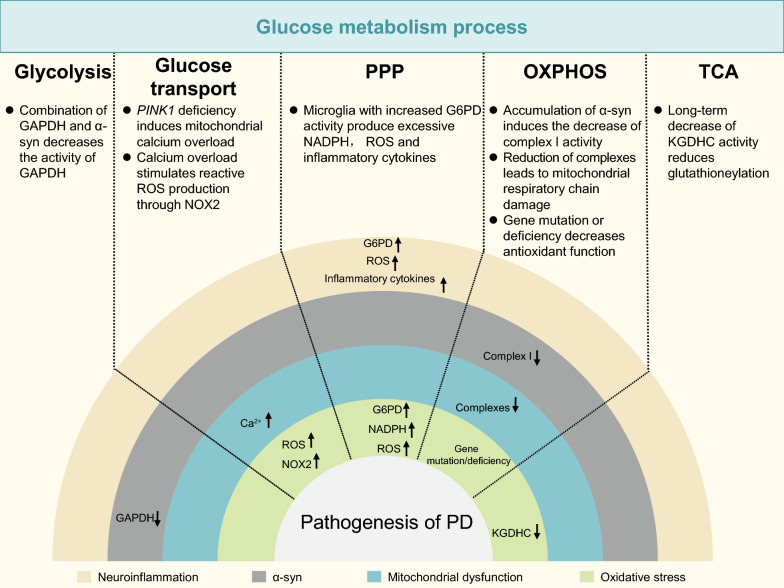
The relationship between glucose metabolism and PD pathogenesis. PPP, pentose phosphate pathway; OXPHOS, oxidative phosphorylation; TCA, tricarboxylic acid; α-syn, α-synuclein; GAPDH, glyceraldehyde-3-phosphate dehydrogenase; NOX2, NADPH oxidase 2; G6PD, glucose-6-phosphate dehydrogenase; ROS, reactive oxygen species; *PINK,* PTEN induced putative kinase 1

Diabetes mellitus (DM) and PD share some pathophysiological mechanisms, such as oxidative stress, mitochondrial dysfunction, neuroinflammation, insulin resistance, and protein misfolding [59]. In the following, we summarize the pathophysiological mechanisms of PD in relation to glucose metabolism impairment (Fig. 3).

#### Glucose transport

Glucose enters the brain through the blood–brain barrier and is transported across cell membrane via a specialized transport system, which includes both Na-independent glucose transporters (GLUT) and Na-dependent sodium-glucose cotransporters (SGLT). Glucose transporters such as GLUT1, GLUT3, GLUT4, GLUT8, SGLT1 and SGLT6 have been found to be expressed in various brain regions [60].

Mutations in PTEN-induced kinase 1 (*PINK1*) cause autosomal recessive PD [61]. PINK1 is a mitochondrial kinase of unknown function. In 2009, a study found that PINK1 regulates calcium outflow from mitochondria through the mitochondrial Na^+^/Ca^2+^ exchanger. PINK1 deficiency causes mitochondrial calcium accumulation, resulting in mitochondrial calcium overload. The calcium overload stimulates the production of reactive oxygen species (ROS) through NADPH oxidase (NOX2), which inhibits glucose transporters and reduces substrate transfer, resulting in impaired respiration [62]. In recent years, research has shown that the GLUT1 level is reduced in the striatum of a mouse model of PD induced by 1-methyl-4-phenyl-1,2,3,6-tetrahydropyridine (MPTP). In addition, upregulation of GLUT3 protects against MPP^+^-induced toxicity in cell lines [63]. Similarly, the pesticide paraquat (PQ) stimulates glucose transport and promotes translocation of GLUT4 and SGLT1 to the plasma membrane. Moreover, only inhibition of GLUT-like transporters with STF-31 or ascorbic acid (a competitive inhibitor of GLUT-like glucose transport) provides protection against PQ toxicity [64]. SGLT6 and GLUT8 (and possibly GLUT4) appear to be the primary glucose transporters in the SN [65, 66]. These findings underscore the crucial role of glucose transport and metabolism in dopaminergic cell death and the development of PD.

#### Glycolysis

Glycolysis is the process by which glucose is converted to pyruvate or lactic acid, resulting in the production of ATP and metabolites that provide energy for life activities. The connection between glycolysis and PD involves the enzyme phosphoglycerate kinase 1 (PGK1), the first ATP-producing enzyme in the glycolytic pathway, encoded by the *PGK1* gene. In a previous report, PGK activity was significantly decreased in muscle of a young man with severe PD syndrome, although with no evidence of hemolytic anemia. Molecular analysis identified p.T378P mutation in the *PGK1* gene in this patient. This suggests a relationship between PGK deficiency and PD, although *PGK1* sequencing in a cohort of idiopathic PD cases is needed [67]. In 2017, Sakaue et al. reported that a boy with PGK-1 deficiency and his mother, a carrier of heterozygous PGK-1 mutation, both exhibited early-onset PD syndrome. This was the first report describing PD syndrome in carriers of PGK-1 deficiency. The *PGK1* gene is located on the X chromosome, within the confirmed susceptibility region for PD known as PARK12, suggesting that *PGK1* may be directly involved in the disease [68]. Interestingly, terazosin (TZ) has been shown to enhance PGK1 activity, thereby stimulating glycolysis and increasing cellular ATP levels. In various PD models, including toxin-induced and inherited forms in mice, rats, fruit flies, and induced pluripotent stem cells, TZ increases brain ATP levels, slowing or preventing neuronal loss. Additionally, data from the Parkinson's Progression Markers Initiative and the IBM Watson/Truven Health Analytics Market Scan Database showed that individuals with PD who used TZ and related drugs experienced slower disease progression and fewer PD-related complications, and PD-free individuals using TZ and related drugs had a lower risk of PD diagnosis [69]. Thus, enhancing PGK1 activity and boosting glycolysis may offer a promising approach to slowing neurodegeneration in PD.

Glyceraldehyde-3-phosphate dehydrogenase (GAPDH) is another key enzyme in glycolysis, catalyzing the conversion of glyceraldehyde-3-phosphate to 1,3-bisphosphoglycerate. Beyond its metabolic role, GAPDH is a multifunctional protein with activities in the cytoplasm, membrane, and nucleus. GAPDH in the nucleus is involved in apoptosis, particularly neuronal apoptosis. In PD cell models, GAPDH co-localizes with α-syn in Lewy bodies, accompanied by reduced GAPDH activity [70]. The binding of α-syn to GAPDH with partially oxidized active site cysteines leads to the inactivation of the enzyme. This interaction also results in formation of a complex between GAPDH and monomeric α-syn, preventing the amyloid conversion of α-syn, which further promotes GAPDH inactivation and inhibits glycolysis [71, 72]. Additionally, glycosylation of α-syn has been shown to increase its binding to GAPDH, leading to greater enzyme inactivation and a further reduction in glycolysis [73]. However, current research on the relationship between the two is limited to in vitro models. Whether there is a similar mechanism in PD patients needs further exploration.

**Fig. 4:**
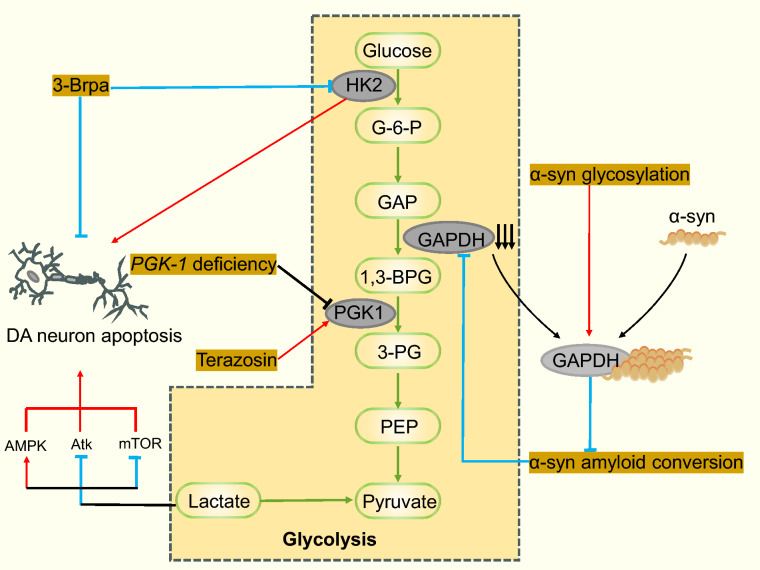
Pathological changes of glycolysis in PD. Expression of hexokinase 2 (HK2) and the lactate levels are markedly increased in PD condition. 3-Bromopyruvic acid (3-Brpa), an HK2 inhibitor, suppresses apoptosis of DA neurons. Elevated lactate levels lead to increased AMPK (adenosine monophosphate-activated protein kinase) activity, inhibition of Akt (serine/threonine kinase 1) and mTOR (mammalian target of rapamycin) phosphorylation, promoting DA neuron apoptosis. Phosphoglycerate kinase 1 (PGK1) deficiency leads to a decrease in PGK1 activity, and trazodone can enhance PGK1 activity to exert a therapeutic effect. The binding of α-syn to glyceraldehyde-3-phosphate dehydrogenase (GAPDH) leads to inhibition of GAPDH activity. Their conjugates can inhibit the amyloid conversion of α-syn, which further promotes GAPDH inactivation and inhibits glycolysis. In addition, glycosylation of α-syn can increase its binding to GAPDH, further inhibiting the activity of GAPDH. G6PD, glucose-6-phosphate dehydrogenase; GAP, glyceraldebyde-3-phosphate; 1,3-BPG, 1,3-bisphosphoglycerate; 3-PG, 3-phosphoglycerate; PEP, phosphoenolpyruvate. Red arrows represent promotion, and blue lines represent inhibition

Lactic acid is a by-product of glycolysis. Patients with advanced PD have abnormally elevated CSF levels of lactic acid [74]. One study found that the expression of hexokinase 2 (HK2) and the lactate levels are markedly increased in the SN pars compacta of MPTP-treated mice and in MPP-treated SH-SY5Y cells. Exogenous lactate treatment leads to the apoptosis of SH-SY5Y cells. Intriguingly, 3-bromopyruvic acid, an HK2 inhibitor, suppresses lactate production and apoptosis of DA neurons both in vivo and in vitro. The 3-bromopyruvic acid treatment also markedly improves the motor behavior of MPTP-treated mice in pole test and rotarod test. Mechanistically, lactate increases the activity of adenosine monophosphate-activated protein kinase (AMPK) and suppresses the phosphorylation of serine/threonine kinase 1 (Akt) and mammalian target of rapamycin (mTOR) to prompt the apoptosis of DA neurons in PD [75] (Fig. 4).

#### TCA

Accumulating evidence shows that TCA is involved in PD pathogenesis. Deficiency of PD-related gene *PINK1* triggers hypoxia-inducible factor-1α (HIF1α) stabilization both in vivo and in vitro. The stabilization of HIF1α leads to the up-regulation of HIF1 target pyruvate dehydrogenase kinase-1 (PDK1), which phosphorylates and inhibits activity of pyruvate dehydrogenase, an enzyme involved in TCA. Therefore, this may reduce the flow of TCA and ultimately impair energy supply. Meanwhile, HIF1α stimulates glycolysis to sustain cell proliferation [76].

The α-ketoglutarate dehydrogenase complex (KGDHC) is a three-enzyme complex that catalyzes the oxidation of α-ketoglutarate to succinic acid and is a rate-regulating enzyme for the TCA cycle. The toxin MPP^+^ can affect TCA by inhibiting mitochondrial KGDHC in PD animal models. In 1994, Mizuno et al. found reduced immunostaining of KGDHC in many melanized neurons in PD compared with the control group, and these neurons were more common in the lateral one-third of the SN. In addition, the decrease of KGDHC immunostaining was roughly related to the severity of degeneration. This was the first discovery of the role of KGDHC in the progression of PD [77]. Interestingly, in 2003, Gibson et al. reported that the specific decrease of KGDHC occurs even in pathologically unaffected areas in PD, and the decrease is unlikely to be a non-specific result of neurodegeneration. Therefore, reductions in the activity of this enzyme are widespread in the brain, which may make vulnerable areas susceptible to further damage [78]. Genetically, in 1998, Kobayashi et al. found that the frequency of KGDHC E2 (dihydrolipoamide succinyltransferase) genotypes containing allele 2 was significantly higher in PD group than in control group. It is suggested that the genetic variation of E2 gene itself or very close to the gene is one of the genetic risk factors of PD, implying an important role of KGDHC in PD [79].

Further experimental studies suggest that KGDHC not only limits the rate of NADH production and substrate-level phosphorylation, but is also a source of ROS in mitochondria [80]. Subsequently, a study found that reducing KGDHC with adeno virus diminished neurogenesis and increased oxidative stress in vivo. Glutathionylation of KGDHC can be viewed as an antioxidant response protecting the enzyme from oxidative damage. In vitro, short-term reductions of KGDHC are protective, increasing glutathionylation and enhancing the ability of cells to diminish the ROS from added oxidants. However, long-term reductions weaken the ability to diminish ROS and reduce glutathionylation. Moreover, increasing KGDHC enhances the ability of cells to diminish externally added ROS [81].

#### OXPHOS

Multiple lines of evidence from toxicological, molecular, and genetic studies have linked mitochondrial OXPHOS dysfunction to PD. In 1991, Gerlach et al. reported that the selective blockade of respiratory chain complex I by MPP^+^ induces dopaminergic neuronal degeneration and PD symptoms, suggesting that defects in the respiratory chain play a role in the pathogenesis of PD [82]. In the following years, studies revealed a significant decrease of complex I activity in the SN of patients with idiopathic PD [83, 84], as well as reduced activity of complexes I and IV in peripheral blood [85]. Interestingly, both in vivo and in vitro studies have shown that the accumulation of α-syn in the mitochondria of human DA neurons leads to decreased complex I activity, increased production of ROS, reduced substrate-specific respiration, and increased mitophagy [86, 87]. Moreover, several PD-associated genes, such as *PINK1, Parkin, DJ-1/PARK7,* the Leucine-rich repeat kinase2 (*LRRK2*), the Coiled-Coil-Helix-Coiled-Coil-Helix Domain Containing 2 (*CHCHD2*), *VPS35* and the tumor necrosis factor receptor associated protein 1 (*TRAP1*), have been shown to be involved in OXPHOS regulation [88] (Table 2).

**Table 2:** Effects of PD-associated genes on the respiratory chain

Gene	Gene locus	Inheritance	Model	Function	Complexes targeted
*PINK1*	1p36.12	AR	*Pink1-*deficient *Drosophila* and mouse models	*PINK1* deficiency disrupts complex I function, leading to mitochondrial membrane depolarization, increasing sensitivity to apoptotic stress and synaptic transmission defects [90]	I [92]
			Primary cortical neurons from *Pink1*-deficient mouse models	Controls the phosphorylation of FKBP5 to regulate the phosphorylation of Akt, thereby preventing complex I inhibition by inhibitors such as MPP + [91]	
*Parkin*	6q26	AR	*Parkin*-deficient mouse models	*Parkin* deficiency reduces respiratory capacity of striatal mitochondria and levels of proteins involved in the protection against oxidative stress [96]	I, IV [96]
			*Parkin*-mutant iPSC neurons	*Parkin* mutation leads to increased oxidative stress, abnormal mitochondrial morphology and impaired mitochondrial homeostasis [97]	
*DJ-1*	1p36.23	AR	*DJ-1* deficient mice or cell models	*DJ-1* deficiency can lead to decreased motor activity, increased striatal denervation, loss of dopaminergic neurons, and increased sensitivity to oxidative damage [98]	I [99]
*LRRK2*	12q12	AD	G2019S-LRRK2 transgenic mice	Increases vulnerability to stress injury [101]	I [102]
*CHCHD2*	7p11.2	AD	Fibroblasts of PD patients	The *CHCHD2* mutation leads to fragmentation of the mitochondrial network [103]	I, IV, V [104]
*VPS35*	16q11.2	AD	Fibroblasts of PD patients	The *VPS35* D620N mutation leads to decreased enzyme activity of complexes I and II and respiratory defects [105]	I, II [106]
*TRAP1*	16p13.3	AR	*TRAP1*-deficient *Drosophila* models	*TRAP1* deficiency results in decreased mitochondrial function and increased sensitivity to stress [107]	I, II, IV [107, 109, 110]
			*TRAP1*-deficient cell models	TRAP1 inhibits the activity of mitochondrial c-Src by binding to mitochondrial c-Src and preventing its self-activation by phosphorylation, thus playing a role in mitochondrial respiration [111]	

AD, autosomal dominant; AR, autosomal recessive

Mutations in *PINK1* have been identified in patients with juvenile Parkinsonism [89]. Pink1 encoded by the *PINK1* gene is a key mitochondrial kinase implicated in PD. Numerous studies have shown that PINK1 loss-of-function impairs both mitochondrial autophagy and OXPHOS. Studies in *Pink1*-deficient *Drosophila* and mouse models demonstrated that an early consequence of *Pink1* deficiency is the disruption of complex I function, leading to mitochondrial membrane depolarization, increased sensitivity to apoptotic stress, and synaptic transmission deficits [90]. Another study using primary cortical neurons cultured from *Pink1*-deficient mice revealed that FKBP5 can reduce Akt phosphorylation and promote MPP^+^-mediated neuronal death in the absence of *Pink1* [91]. Thus, the loss of PINK1 reduces complex I activity, resulting in impaired mitochondrial respiration [90, 92].

Mutations in the *Parkin* gene are a major cause of early-onset autosomal-recessive familial PD and isolated juvenile-onset PD [93]. Parkin functions as an E3 ubiquitin ligase, playing a neuroprotective role in maintaining mitochondrial metabolism and regulating the ubiquitin–proteasome system [94]. In this system, Parkin is crucial for the ubiquitin-mediated degradation of misfolded or damaged proteins and the removal of dysfunctional mitochondria through mitophagy [95]. Interestingly, Parkin-deficient mice demonstrated a reduction of several subunits of complexes I and IV, decreased mitochondrial respiratory capacity in the striatum, and lower levels of proteins involved in protection against oxidative stress [96]. However, since animal models do not fully recapitulate the pathophysiology of human PD, a study employed neurons derived from induced pluripotent stem cells from patients with *Parkin* mutations. The study found increased oxidative stress, abnormal mitochondrial morphology, and impaired mitochondrial homeostasis in these neurons [97].

DJ-1/PARK7 plays a key role in transcriptional regulation and protection against oxidative stress. *DJ-1*-deficient mice exhibit reduced locomotor activity when challenged with amphetamine, along with increased striatal denervation and DA neuron loss induced by MPTP. Additionally, *DJ-1*-deficient embryonic cortical neurons show increased sensitivity to oxidative damage. Restoration of DJ-1 expression in *DJ-1*-deficient mice or cells through adenoviral vector delivery mitigated these phenotypes [98]. DJ-1 directly binds to the nuclear subunit of mitochondrial complex I, as well as the mitochondrial DNA-encoded subunits NDUFA4 and ND1, and co-localizes with complex I. In *DJ-1*-knockout NIH3T3 and HEK293 cells, the complex I activity is significantly reduced [99].

Mutations in the *LRRK2* gene, particularly the most common Gly2019Ser mutation, are seen in patients with autosomal-dominant PD and patients with apparently sporadic PD, who are clinically indistinguishable from patients with idiopathic PD [100]. Cortical neurons from G2019S-*LRRK2* mice are more vulnerable to stress injury, and exposure to subtoxic doses of MPTP can cause severe motor dysfunction, selective loss of DA neurons, and increased astrocyte activation [101]. Furthermore, patients with *LRRK2* gene mutations have reduced activity of complex I in vivo [102], and Lrrk2 in mice protects complex I from inhibitors such as MPP^+^ [101].

CHCHD2, a mitochondrial stress response protein, is involved in OXPHOS and the maintenance of cristae morphology [103]. Interestingly, a study found that mutations in *CHCHD2* in fibroblasts from PD patients lead to fragmentation and reduced OXPHOS activity of complexes I and IV [104].

VPS35 regulates mitochondrial fusion protein 2 (Mfn2) by controlling the transport of mitochondrial ubiquitin ligase 1 (MUL1) to the mitochondrial membrane, promoting mitochondrial fusion, which in turn increases ATP production and reduces mitophagy [105]. The *VPS35* D620N mutation elevates MUL1 protein levels, leading to reduced enzyme activity of complexes I and II, as well as respiratory defects in fibroblasts from PD patients [106].

Although *TRAP1* is not a familial PD gene, it is closely associated with familial PD and the OXPHOS-related pathway. TRAP1 is a cellular substrate for the PINK1 kinase, and mutations in the *TRAP1* gene in *Drosophila* models lead to significantly reduced dopamine levels and decreased complex I activity. Deletion of *TRAP1* leads to impaired mitochondrial function and increased sensitivity to stress [107]. Interestingly, TRAP1 appears to act downstream of PINK1 to prevent mitochondrial dysfunction associated with PD pathogenesis [108], a mechanism also validated in *Drosophila* models [107]. Meanwhile, mitochondrial c-Src, a tyrosine kinase, has been shown to phosphorylate and promote the activity of several components of the mitochondrial respiratory chain, including complex II [109] and complex IV [110]. TRAP1 inhibits mitochondrial c-Src activity by binding to it and preventing its auto-activation by phosphorylation, thereby exerting some of its effects on mitochondrial respiration through this kinase [111].

#### Gluconeogenesis

The relationship between gluconeogenesis and PD has been studied. One study demonstrated that gluconeogenic intermediates, such as pyruvate, malate, and phosphoenolpyruvate (PEP), are neuroprotective against MPP^+^ toxicity. Glycolytic intermediates are shown to increase intracellular ATP levels, promoting anaerobic glycolysis by enhancing the availability of anaerobic substrates [112]. Similarly, another study found that MPTP administration suppresses expression of proteins involved in glycolysis and gluconeogenesis [113]. Additionally, a recent study revealed an increased contribution of gluconeogenesis to the total glucose production in patients with idiopathic PD (*n* = 33) compared to healthy controls (*n* = 13). These findings suggest a potential link between gluconeogenesis and PD [114]. However, due to the lack of longitudinal studies, the underlying pathological mechanisms remain to be further explored.

#### PPP

A study using metabolic tracing revealed that the ^13^C-labeled glucose is more likely to enter the PPP pathway under high glucose stimulation [115]. The PPP converts glucose-6-phosphate to pentose and produces ribose-5-phosphate and NADPH. Glucose-6-phosphate dehydrogenase (G6PD), a NADPH-generating enzyme, is the rate-limiting enzyme for the PPP. Glucose metabolism in neurons is primarily directed to the generation of reducing equivalents via the PPP to support antioxidant defenses.

In 2005, Abraham et al. reported that the G6PD activity is significantly lower in PD patients than in the control group [116]. Subsequently, a postmortem human brain tissue study showed increased NADPH production in the putamen, cortex, and cerebellum of late-stage PD cases, while G6PD was decreased in the putamen of early-stage PD and in the cerebellum of early and late-stage PD. However, the levels of NADPH and G6PD in the SN (the main affected area of PD) were not examined in the study [117]. However, another finding suggested that G6PD expression and activity are elevated in the SN both in vitro and in vivo in PD, and that inhibition of G6PD and knockdown of microglial G6PD significantly attenuated LPS-induced dopaminergic neurodegeneration [118]. In addition, up-regulation of NOX2 has been detected in the SN of PD patients and a mouse model of PD, and that over-activated NOX2 is a major source of oxidative stress under inflammatory conditions [119]. NOX2 and nitric oxide synthase use NADPH as a cofactor to produce free radicals [120]. Furthermore, peripheral blood monocytes of human subjects with G6PD deficiency show reduced secretion of inflammatory cytokines such as TNFα and IL-1β [121]. Mechanistically, microglia with elevated G6PD activity produce excess NADPH, which provides abundant substrate for overactivated NOX2. This results in excessive production of ROS, leading to oxidative stress and the onset of neurodegenerative pathology. At the same time, microglia secrete inflammatory cytokines to produce inflammatory responses, further exacerbating the neurotoxicity [118]. Increased G6PD caused by PQ exposure increases PQ toxicity and NADPH reduction equivalents, and induces oxidative stress and cell death [122]. Interestingly, there are also reports claiming that increase of G6PD activity in DA neurons alleviates MPTP-induced neurotoxicity in mice, and aged animals also demonstrate the neuroprotective effect of G6PD [123]. The heterogeneity of G6PD activity across the above studies may be due to the differences in sample size, sample types, measurement methods/sensitivity, and disease stage/severity. Therefore, the specific mechanism of G6PD in the development of PD needs to be further explored.

PPP activity in astrocytes is 5–7-fold greater than that in neurons, and glutathione synthesis is more active in astrocytes than in neurons. Astrocytes provide neurons with reduced glutathione (GSH) through PPP, and GSH may act as an independent ROS scavenger or a substrate of antioxidant enzymes [124]. Neurons express minimal levels of the system x_c_^−^ cystine/glutamate antiporter, rendering them unable to efficiently capture cystine from the extracellular milieu. Cystine is the oxidized form of cysteine and is indispensable for the synthesis of GSH. Thus, neurons are dependent on the release of GSH by astrocytes [125]. The brain with high cerebral metabolic rate, unsaturated fatty acids and iron content, and an inefficient antioxidant system, is highly susceptible to oxidative stress [125]. Interestingly, PD patients show significantly reduced glutathione levels in the SN [126], suggesting a compromised PPP flux as a factor of PD oxidative stress. However, it has also been reported that the PPP flux decrease may be an early event in the pathogenesis of sporadic PD, because in the later stages of PD, PPP flux is up-regulated in an attempt to counteract persistent oxidative stress [117].

## Potential PD CSF biomarkers related to glucose metabolism

CSF is a reliable biomarker source for neurodegenerative diseases. Unlike plasma, CSF is not separated from the brain by the blood–brain barrier. Recent studies have found many components in the CSF that contribute to early diagnosis of PD and observation of disease development. Decreased dopamine metabolites [127], 5-hydroxytryptamine metabolites [128], β-glucocerebrosidase [129], total α-syn [130], and circulating free mitochondrial DNA [132], as well as increased neurofilament light chain protein [131] and miRNAs [133], have been detected in the CSF in PD.

There are also PD biomarkers related to abnormal glucose metabolism in the CSF. Glucose [134] and lactic acid [135] are increased in the CSF of PD patients, indicating that glucose metabolism does play a role in PD. In addition, advanced glycation end-products (AGEs) are irreversible products from glycosylation reaction. Previous studies have detected increased AGE proteins and AGE receptors in the SN of PD patients [136]. Furthermore, elevated fructose levels in the CSF may also reflect pathological accumulation of AGEs in PD patients, which cause oxidative stress and subsequent cell dysfunction through AGE receptors [134, 137]. Likewise, reduced dehydroascorbic acid (an oxidized form of ascorbic acid) combined with increased threonic acid (a major breakdown product of ascorbic acid) levels in the CSF indicates a change in oxidative stress response: dehydroascorbic acid has neuroprotective effects against free radicals in the brain, while threonic acid may interfere with the metabolism of ascorbic acid [137]. Interestingly, mannose binding lectin (MBL), a lectin involved in the recognition of infectious agents by the innate immune system, is significantly down-regulated in PD patients. Some studies have proposed the hypothesis that the increase of mannose level in the CSF of PD patients may be directly related to the decreased MBL level that leads to decreased mannose binding, thus increasing the accumulation of free mannose in the CSF. However, further clarification is needed to verify this hypothesis [134]. Compounds related to glucose metabolism in the CSF may serve as potential biomarkers for the diagnosis of PD. Nevertheless, these studies involve small sample sizes, and the identification of reliable biomarkers requires larger sample cohorts (Table 3).

**Table 3:** **CSF** compounds related to glucose metabolism impairment in PD

Reference	Sample size	Compound/change	Findings
Trezzi et al. [134]	PD patients: 44 Control individuals: 43	Glucose/increase	Abnormal glucose metabolism exists in PD
Liguori et al. [135]	Drug-naive PD patients: 101 Control individuals: 60	Lactate acid/increase	Abnormal glucose metabolism exists in PD
Dalfó et al. [136]	Patients with neuropathologically verified PD-related changes: 4 Age matched controls: 3	AGEs/increase	Oxidative stress exists in PD
Barabás et al. [137]	Early-stage sporadic PD patients without treatment: 44 Age- and gender-matched healthy controls: 43	Fructose/increase Threonic acid/increase Dehydroascorbic acid/decrease Mannose/increase	Glycosylation causes oxidative stress and subsequent cellular dysfunction Neuroprotective effects of ascorbic acid on free radicals in the brain The increase of free mannose level in CSF of PD patients may be directly related to the decrease of MBL level

PD, Parkinson's disease; AGEs, advanced glycation end products; MBL, mannose-binding lectin; CSF, cerebrospinal fluid

## Glucose metabolism and PD drugs

Currently, the commonly used PD therapeutic drugs also seem to regulate glucose metabolism, which provides evidence for the connection between PD and glucose metabolism.

### Levodopa

Current PD treatment is mainly based on dopamine replacement therapy, as exogenous dopamine and other catecholamines cannot cross the blood–brain barrier. Levodopa is a direct precursor to dopamine and a suitable prodrug because it can cross the blood–brain barrier and convert to dopamine. It remains the gold-standard therapy for PD, especially in the early stages. Islet is the site of dopamine synthesis [138–140]. Recent studies have shown that levodopa and dopamine suppress glucose-dependent insulin secretion by reducing the frequency of intracellular calcium current oscillation [141]. The dopaminergic regulation of insulin secretion is a possible link between PD and T2DM [142].

### Bromocriptine

As a dopamine agonist, bromocriptine was first introduced in 1974 as an adjunct therapy for routine levodopa treatment in patients with motor fluctuations due to PD. Bromocriptine activates presynaptic dopamine receptors in the basal ganglia, hypothalamus, and midbrain limbic system, thereby restoring both motor and non-motor deficits in PD [143, 144]. Additionally, bromocriptine enhances glycemic control and glucose tolerance in obese type 2 diabetic patients, as well as in obese non-diabetic animals and humans. Mechanistically, it reduces blood glucose and improves glucose homeostasis by activating D2 receptors and blocking D1 receptors. Furthermore, bromocriptine directly activates α2-adrenergic receptors and inhibits glucose-stimulated insulin secretion in pancreatic β cells [58, 145]. Therefore, bromocriptine has dual actions in patients with PD who also have glucose metabolism abnormalities.

## Potential therapeutic drugs/compounds

### Drugs/compounds targeting glucose metabolism

**Fig. 5:**
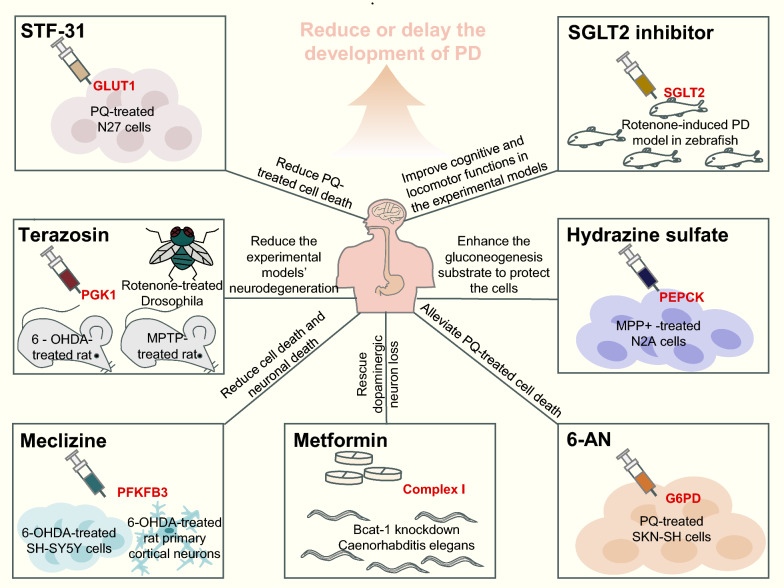
Drugs targeting glucose metabolism in PD. Some enzymes involved in glucose metabolism are potential targets for PD treatment. The figure illustrates some PD drugs that are revealed to act through these targets (GLUT1, PGK1, PFKFB3, SGLT2, PEPCK, and G6PD) in experimental models. PGK1, phosphoglycerate kinase 1; PFKFB3, fructose-2,6-bisphosphatase 3; G6PD, glucose-6-phosphate dehydrogenase; PEPCK, PEP carboxykinase; 6-AN, 6-aminonicotinamide; SGLT2, sodium-glucose cotransporter 2; MPTP, 1-methyl-4-phenyl-1,2,3,6-tetrahydropyridine; MPP, 1-methyl-4-phenylpyridinium; 6-OHDA, 6-hydroxydopamine; PQ, paraquat

Based on the previous section, we can hypothesize that compounds targeting glucose metabolism may have potential therapeutic benefits in PD (Fig. 5).

Inhibition of GLUT-like glucose transport with STF-31 (a GLUT1 inhibitor) significantly reduces the PQ-induced death of N27 dopaminergic cells [64]. In addition, SGLT2 inhibitors, as current drugs for the treatment of T2DM, have a potential impact on PD. The SGLT2 inhibitor empagliflozin has a beneficial effect in rotenone-induced zebrafish model of PD [146]. Similarly, in real-world settings, a Korean cohort study involving 358,862 participants showed that SGLT2 inhibitors reduce the risks of dementia and PD in T2DM patients [147]. Additionally, another study from the United States found that SGLT2 inhibitors are associated with a significantly reduced risk of PD in elderly T2DM patients [148]. Besides, in a population-based study, the risk of dementia is reduced by 11% in patients using SGLT2 inhibitors compared to non-users [149]. SGLT2 inhibitors selectively target SGLT2 on the surface of the proximal renal tubular lumen, inhibiting glucose reabsorption and promoting urinary glucose excretion [150]. This reduces the levels of glucose and glycated hemoglobin in the plasma of T2DM patients [151], resulting in decreased insulin levels and increased glucagon release, thereby promoting the production of ketones [152]. Compared with glucose, ketones are more efficiently utilized by neurons, astrocytes and oligodendrocytes to provide more effective energy supply for brain cells [153]. Therefore, SGLT2 inhibitors can support neuronal survival by optimizing brain glucose metabolism and supplementing energy supply.

TZ slows or prevents neuronal loss, increases dopamine levels and partially restores motor function in MPTP mouse model, 6-OHDA rat model, and rotenone *Drosophila* model of PD, by potentiating PGK1 activity, enhancing glycolysis, and increasing cellular ATP levels. Consistently, individuals taking TZ have slower disease progression, reduced PD-related complications and decreased frequency of PD diagnosis [69].

Meclizine activates phosphofructokinase by increasing the level of fructose-2,6-bisphosphatase 3 in 6-OHDA-induced SH-SY5Y cells and rat primary cortical cultures, further enhancing glycolysis and preventing cell death [154]. 6-Aminonicotinamide reduces PQ-induced death of SKN-SH cells by inhibiting the PPP pathway through its action on 6-phosphogluconate dehydrogenase production and G6PD [122]. An early study reported that gluconeogenic metabolic intermediates (PEP, pyruvic acid, and malic acid) can elevate intracellular ATP levels and are neuroprotective against MPP^+^ in N2A cells. Interestingly, impeding gluconeogenesis by hydrazine sulfate, which inhibits PEP carboxykinase, further improves the protection against MPP^+^ [112].

In a longitudinal study conducted in the United States in 2019, over 5500 veterans (about 60 years old) with T2DM were followed up for 5 years to explore the effect of metformin on neurodegenerative disease. The results pointed out that metformin treatment for more than 4 years can significantly reduce the risk of PD (HR 0.19, 95% CI 0.12–0.31) [155]. In the *C. elegans* model of PD with RNAi-mediated *bcat*-1 knockdown, metformin reduced mitochondrial respiration, rescued neuronal viability, and significantly improved motor function, possibly through inhibiting complex I [156]. Thus, metformin may be beneficial by providing mild inhibition of complex I, because the reduced ATP/ADP ratio activates the AMPK signaling pathway, thereby promoting autophagy-mediated degradation of misfolded proteins and dysfunctional organelles [157]. On the contrary, excessive inhibition of complex I is sufficient to trigger the PD phenotype [158]. Interestingly, a recent paper showed that low metformin concentration may stimulate mitochondrial respiration and complex I activity in mice, rather than inhibiting it [126]. Therefore, it is necessary to further explore the precise metformin doses required to change the AMP/ATP ratio and activate AMPK, in order to fully understand the effect of metformin on complex I (Table 4).

**Table 4:** Drugs/compounds targeting glucose metabolism

Drug	Model	Target	Effect on target	Pathway	Experimental finding	Clinical efficacy	Reference
GLUT1 inhibitor (STF-31)	PQ-induced N27 cell model	GLUT1	Inhibition	Glucose transport	Reduces PQ-induced cell death	–	[64]
SGLT2 inhibitor (empagliflozin)	Rotenone-induced zebrafish model of PD	SGLT2	Inhibition	Glucose transport	Improves cognitive and locomotor functions of rotenone-treated zebrafish	Reduces the risk of PD	[146–148]
Terazosin	MPTP-induced rat model 6-OHDA-induced rat model Rotenone-induced *Drosophila* model	PGK1	Activation	Glycolysis	Ameliorates neurodegeneration	Slows down disease progression and reduces PD-related complications and PD diagnosis frequency	[69]
Meclizine	6-OHDA-treated SH-SY5Y cells and rat primary cortical neurons	PFKFB3	Activation	Glycolysis	Reduces neuronal and cell death	–	[154]
6-Aminonicotinamide	PQ-induced SKN-SH cell model of PD	G6PD	Inhibition	PPP	Alleviates PQ-induced cell death	–	[122]
Hydrazine sulfate	MPP^+^-treated N2A cells	PEPCK	Inhibition	Gluconeogenesis	Increases the level of the gluconeogenesis substrate to protect the cells	–	[112]
Metformin	Bcat-1-knockdown *C. elegans*	Complex I	Inhibition	OXPHOS	Rescues the loss of dopaminergic neurons	Reduces the risk of PD	[155–158]

GLUT1, glucose transporter 1; SGLT2, Na-dependent sodium-glucose cotransporters 2; PGK1, phosphoglycerate kinase 1; PFKFB3, Fructose-2,6-bisphosphatase 3; G6PD, glucose-6-phosphate dehydrogenase; PEPCK, PEP carboxykinase; MPTP, 1-methyl-4-phenyl-1,2,3,6-tetrahydropyridine; MPP, 1-methyl-4-phenylpyridinium; PQ, paraquat; 6-OHDA, 6-hydroxydopamine; PPP, Pentose phosphate pathway; OXPHOS, oxidative phosphorylation

### Drugs associated with insulin signaling

Forkhead box O (FOXO) is a component of the insulin and insulin-like growth factor signaling pathway. Dysfunction of this pathway is the main cause of T2DM [159]. Expression of *FOXO1* gene is significantly increased in PD [160]. dFOXO is the only homologue of FOXO in *Drosophila*. Overexpression of dFOXO has been shown to protect DA neurons in *Drosophila Pink1*-null mutants [161], while other studies have shown that dFOXO-induced apoptosis mediates DA neuron death in *Drosophila DJ-1β* loss-of-function mutants [162] and *dLRRK* mutants [163]. These results indicate the role of FOXO factors in the pathogenesis of sporadic or familial PD. The discrepancy of results may depend on FOXO activity level and the genetic background [164]. More studies are needed to better understand the role of FOXO in the pathogenesis of neurodegenerative diseases and its potential as a therapeutic target for PD treatment.

Glucagon-like peptide-1 (GLP-1) is an incretin hormone that promotes healthy insulin signaling, regulates blood glucose levels, and inhibits appetite. Therefore, GLP-1 receptor agonists (GLP-1RAs) were first used to treat type 2 diabetes. In 2002, Perry et al. found that GLP-1 can reverse or halt neurodegenerative process, and proposed for the first time the therapeutic potential of GLP-1 in PD [165]. Subsequent studies have found that GLP-1 can reduce the risk of PD in type 2 diabetes [166]. Most recently, a 52-week phase 2 clinical trial of liraglutide (a GLP-1RA) in PD patients found that liraglutide treatment improves non-motor symptoms, mobility, and quality of life with safety and good tolerance [167]. In addition, in a two-stage, double-blind, randomized, placebo-controlled trial, lixisenatide treatment in early PD participants resulted in slower progression of movement disorders at 12 months than placebo treatment, but the treatment also caused gastrointestinal side effects [168]. However, due to the small sample size of the study, longer and larger trials are needed to determine the effect of liraglutide and lixisenatide in PD patients. The proven benefits of exenatide, a single GLP-1RA, underscore the enormous potential of anti-insulin resistance therapy to improve outcomes of patients with neurodegenerative diseases. Studies have shown that exenatide treatment promotes insulin signaling pathways in the brain, manifested as increased tyrosine phosphorylation of insulin receptor substrate 1 and increased expression of downstream Akt and phosphorylated mTOR [169]. Additionally, GLP-1 also exerts anti-inflammatory effects through a variety of downstream pathways upon binding to its receptor [170]. Surprisingly, these anti-inflammatory benefits have recently been attributed to central neuronal GLP-1RAs [171], highlighting the potential use of incretin receptor agonists for neurological diseases with inflammatory components. GLP-1RAs provide a safe treatment option for neurodegenerative diseases with both anti-inflammatory and anti-insulin resistance effects. GLP-1RAs have long been evaluated in clinical trials of PD to assess its impact on disease progression, which have yielded very promising results [172]. However, with the availability of single, double and triple GLP-1RAs, further exploration is needed to determine the most appropriate treatment.

Surprisingly, abnormal sphingolipid metabolism has been shown to play a key role in the pathogenesis of PD [173]. Lipid metabolomics studies have found significant changes of sphingolipid composition in the plasma of PD patients compared to the control group [174, 175]. In addition, studies have shown that brain insulin resistance is associated with mitochondrial membrane potential depolarization, mitochondrial biogenesis damage, and increased ROS [176]. Mitochondrial dysfunction is the most basic feature of PD pathogenesis. Abnormal sphingolipid metabolism can lead to the occurrence of PD by inducing insulin resistance [177]. An epidemiological meta-analysis reported that lipid-lowering statins are protective against PD symptoms, further supporting this mechanism [178].

In 2005, Liss et al. reported that the selective vulnerability of SN DA neurons in PD is causally related to the activation of ATP-sensitive potassium (K_ATP_) channels in these neurons [179]. Surprisingly, memantine acting on the K_ATP_ channel of SNc DA neurons provides clinical benefits for the treatment of PD [180]. In recent years, studies in PD patients have also shown a role of memantine in slowing the development of the disease [181, 182]. However, memantine does not seem to directly affect K_ATP_ channel activity [183]. It can increase serum insulin and reduce blood glucose levels in diabetic mice [184]. Memantine is an *N*-methyl-*d*-aspartate (NMDA) receptor antagonist. Inhibition of NMDA receptor expressed on islet β cells can inactivate K_ATP_ channels and ultimately improve blood glucose levels in type 2 diabetic mouse models [185]. This may be one of the mechanisms underlying the effect of memantine in PD, as the K_ATP_ channels on hippocampal neurons are composed of the same subunits as those on islet β cells (Kir6.2 and SUR1) [186].

PD is more prevalent in males than in females. The increased risk of PD in postmenopausal women indicates that estrogen (E2) has neuroprotective effects [187, 188]. Oxidative stress is an important pathogenic mechanism of PD. E2 up-regulates the expression of Nrf2 through estrogen receptor α (ERα), and Nrf2 is an important anti-oxidative stress factor in cells [189]. Estrogen-responsive element (ERE) in the Nrf2 promoter region can also be directly activated by ERα to promote Nrf2 transcription [190]. In rodents, ERβ signaling can also exert anti-oxidative stress effects to resist Nrf2 signaling dysfunction [191]. Therefore, estrogen may be a potential therapeutic drug for PD by inhibiting oxidative stress. Another study found that abnormal fluctuations in E2 and ERα lead to disruption of glucose homeostasis in the brain. Specific knockout of ERα gene in mice can induce glucose intolerance and insulin resistance [192]. Similarly, E2 deficiency leads to hypothalamic glucose homeostasis disorder in ovariectomized rats [193]. The mechanism underlying the regulation of glucose homeostasis by E2 signaling is that the ERα-expressing neurons in the ventrolateral subdivision of the ventromedial hypothalamic nucleus can sense glucose fluctuations throughout the body and regulate glucose homeostasis [194]. Therefore, E2 could be potentially used to treat PD by regulating glucose homeostasis. However, as the use of estrogen increases the risk of diseases such as breast cancer, selective estrogen receptor β agonists may be a better choice [195].

## Conclusions and perspectives

The relationship between PD and glucose metabolism impairment is complex. It is not clear whether glucose metabolism is the cause or the result of PD. Glucose metabolism impairment observed in PD patients involves glucose transport, glycolysis, TCA, OXPHOS, PPP and gluconeogenesis. Although the role of glucose metabolism in PD has been widely studied, few studies have explored processes such as TCA and gluconeogenesis. Some potential PD biomarkers related to glucose metabolism have been widely studied and recognized, which may provide clues for clinical diagnosis. In addition, drugs targeting glucose metabolism have the potential to be used for PD treatment, but there are few studies on the treatment of glucose metabolism impairment in PD patients. More studies on the relationships between glucose metabolism impairment and PD are needed to advance neuroprotective therapies and translate them into clinics.

## Data Availability

Not applicable.

## Abbreviations

AGEs: Advanced glycation end products
AMPK: Adenosine monophosphate-activated protein kinase
Akt: Serine/threonine kinase 1
*CHCHD2*: Coiled-Coil-Helix-Coiled-Coil-Helix Domain Containing 2
CSF: Cerebrospinal fluid
ERα: Estrogen receptor α
FDG-PET: Fluorodeoxyglucose positron emission Tomography
FOXO: Forkhead box O
G6PD: Glucose-6-phosphate dehydrogenase
GAPDH: Glyceraldehyde-3-phosphate dehydrogenase
GLUT: Glucose transporters
GLP-1: Glucagon-like peptide-1
GLP-1 RA: GLP-1 receptor agonist
HIF1α: Hypoxia-inducible factor-1α
iRBD: Idiopathic rapid eye movement sleep behavior disorder
KGDHC: α-Ketoglutarate dehydrogenase complex
*LRRK2*: Leucine-rich repeat kinase2
mTOR: Mammalian target of rapamycin
MPTP: 1-Methyl-4-phenyl-1,2,3,6-tetrahydropyridine
MPP +: 1-Methyl-4-phenylpyridinium
NADPH: Nicotinamide adenine dinucleotide phosphate
NOX2: NADPH oxidase 2
OXPHOS: Oxidative phosphorylation
PD: Parkinson's disease
PDSP: PD specific pattern
PDD: PD dementia
PGK1: Phosphoglycerate kinase 1
PPP: Pentose phosphate pathway
PQ: Paraquat
*PINK1*: PTEN induced putative kinase 1
ROS: Reactive oxygen species
SGLT: Sodium-glucose cotransporter
SN: Substantia nigra
T2DM: Type 2 diabetes mellitus
TCA: Tricarboxylic acid
TRAP1: Tumor necrosis factor receptor associated protein 1
VH: Visual hallucinations
6-OHDA: 6-Hydroxydopamine
